# Delusions in Patients with Dementia with Lewy Bodies and the Associated Factors

**DOI:** 10.1155/2018/6707291

**Published:** 2018-05-07

**Authors:** Ray-Chang Tzeng, Ching-Fang Tsai, Ching-Tsu Wang, Tzu-Yuan Wang, Pai-Yi Chiu

**Affiliations:** ^1^Department of Neurology, Tainan Municipal Hospital (Managed by Show Chwan Medical Care Corporation), Tainan, Taiwan; ^2^Division of Endocrinology and Metabolism, Department of Internal Medicine, China Medical University Hospital, Taichung, Taiwan; ^3^Department of Neurology, Show Chwan Memorial Hospital, Changhua, Taiwan

## Abstract

**Objective:**

Delusions are common neuropsychiatric symptoms in patients with dementia with Lewy bodies (DLB). The aim of this study was to investigate the associated factors of delusions in patients with DLB.

**Method:**

A retrospective study of outpatients with DLB registered in a regional hospital's database was performed. The associated factors including cognitive performance, clinical features, vascular risk factors, and neuropsychiatric symptoms between delusional and nondelusional patients with DLB were compared.

**Results:**

Among 207 patients with DLB, 106 (51.2%) were delusional and 101 (48.8%) were not. Delusion of other persons are stealing was the most common symptom (35.3%). The delusional group had a significantly higher diagnostic rate of probable than possible DLB, higher disease severity, poorer cognitive performance, more severe neuropsychiatric symptoms, and higher caregiver burden (all *p* < 0.05). In addition, the delusional group had a significantly lower frequency of diabetes compared to the nondelusional group (odds ratio = 0.28, *p* < 0.001).

**Conclusion:**

Delusion of other persons are stealing was the most common delusional symptom. The patients with DLB who presented with delusions had poorer cognitive function and more severe neuropsychiatric symptoms. A novel finding is that the DLB patients with diabetes had a lower frequency of delusions.

## 1. Introduction

Dementia with Lewy bodies (DLB) is the second most common degenerative dementia. According to the first consensus criteria for the diagnosis of DLB in 1996, it accounts for about 20% of all clinical and autopsy cases of degenerative dementia [1]. In a more recent systemic review of studies on the incidence and prevalence of DLB in 2005, it was reported to account for 0 to 30.5% of all dementia cases [2]. Delusions are among the most common neuropsychiatric features in patients with dementia, especially in those with DLB. Therefore, delusions become one of the supportive features for the clinical diagnosis of DLB [1, 3]. Studies on delusions in dementia have shown that delusions are seldom observed in the predementia stage; however, delusions increase in frequency from the early through the later stages of dementia [4–8].

Clinical studies of delusions in dementia have reported different frequencies and characteristics of delusions among different types of dementia [9]. Psychotic symptoms including delusions and hallucinations have been reported to be significantly more frequent in patients with DLB than in patients with Alzheimer's disease (AD) or other dementia [9–14]. Delusional misidentification is significantly more characteristic of DLB than AD, while paranoid delusions are not specifically associated with DLB [12]. Patients with DLB have more psychotic and mood symptoms; therefore, the carers of patients with DLB experience more stress than those caring for patients with AD and vascular dementia [9].

Pathophysiological studies of delusions in dementia have revealed specific neural substrates that may be associated with delusions in patients with DLB [12, 15–17]. In a study on correlations between cholinergic dysfunction and neuropsychiatric symptoms of dementia, the authors found that defective cholinergic activity in patients with DLB was correlated with hallucinations and delusions [15]. An autopsy study revealed that delusions in DLB are associated with elevated M_1_ binding in Brodmann area 36 [16]. Unlike AD, DLB has been reported to be significantly inversely associated with tangle burden and psychosis [12]. A genetic study reported that the 5-HTTLPR polymorphism is associated with delusions in Lewy body dementias including DLB and Parkinson's disease dementia (PDD) [17].

There is robust evidence of the contribution of vascular risk factors (VRFs) to the incidence and prevalence of AD and vascular dementia (VaD) [18–21]. Diabetes is among the most important VRFs, and a recent meta-analysis reviewed 28 studies and revealed that diabetes has a relative risk (RR) of 1.76 for developing all types of dementia [18]. A case-control study of the risk factors for AD, PD, and DLB by Boot et al. found no association of diabetes with DLB [22]. Some studies demonstrated that most of the risk factors appeared in midlife and that they may increase the risk of dementia later in life [21, 23]. However, associations of VRF with the clinical presentation of dementia have seldom been discussed, and studies on the relationship between VRF and the clinical presentation of DLB even less so [22].

The aim of this study was to investigate factors including clinical features, cognitive performance, neuropsychiatric symptoms, and vascular risk factors between delusional and nondelusional patients with DLB.

## 2. Methods

### 2.1. Database

This is a retrospective study of outpatients with DLB registered in a health system's dementia database. The following information from this database was used for this study:

1. Diagnosis of dementia according to the criteria for primary degenerative dementia in the fourth edition of the Diagnostic and Statistic Manual of Mental Disorders (DSM-IV). Diagnosis of DLB according to the revised consensus criteria for probable or possible DLB developed by the third report of the DLB consortium [3]
2. Age, gender, education, dementia severity, and medications at the time of entry
3. Clinical DLB features including fluctuation, parkinsonism, visual hallucinations, REM sleep behavior disorder (RBD), and severe neuroleptic sensitivity
4. Cognitive performance on the Cognitive Abilities Screening Instrument, Chinese version (CASI C-2.0) with the following domains: long-term memory, short-term memory, attention, mental manipulation, orientation, abstract thinking, language, drawing, and verbal fluency [24]
5. Neuropsychiatric symptoms in the 12-item version of the Neuropsychiatric Inventory (NPI) including delusions, hallucinations, agitation, depression, anxiety, euphoria, apathy, disinhibition, irritation, aberrant motor behavior, night behavior, and eat/appetite behavior on the basis of observations within the past month [25]
6. Clinically relevant vascular risk comorbidities including hypertension, arrhythmia, coronary artery disease, diabetes, hyperlipidemia, and cerebrovascular disease (history of stroke/transient ischemic attack or the diagnosis of vascular encephalopathy in brain imaging)

### 2.2. Assessment of Clinical Features and Diagnosis of DLB

In the dementia clinic, all of the patients and their main caregivers were interviewed by a behavioral neurologist for the assessment of core and suggestive features. Fluctuation was diagnosed when a clinical history of fluctuation in cognition and a Mayo Fluctuation Composite Score (MFCS) > 2 [26] were both present. Visual hallucinations (VHs) were diagnosed when a clinical history of recurrent complex VHs were present. Parkinsonism was diagnosed when at least two of the following were present: bradykinesia, tremor, rigidity, and postural instability. RBD was diagnosed when the minimal criteria for REM sleep behavior disorder according to the International Classification of Sleep Disorders (ICSD) [27] was met. Severe neuroleptic sensitivity was diagnosed when a clinical history was established for both the usage of neuroleptic drugs and an obvious association of adverse events with the neuroleptic drugs. Because dopamine transporter uptake imaging was not available in our hospital until 2010, the suggestive feature “low dopamine transporter uptake in basal ganglia” in the revised consensus criteria could not be evaluated and was thus not included in this study. This may have resulted in a lower diagnostic rate for probable DLB and a higher diagnostic rate for possible DLB.

### 2.3. Assessment of Delusions and Other Neuropsychiatric Symptoms

All of the patients and their main caregivers were interviewed by a trained neuropsychologist for assessment of the NPI domain of delusions, including ratings on eight individual forms of delusions for the past one month. The NPI is a validated, standardized, and widely used instrument that was developed specifically to evaluate the neuropsychiatric symptoms of dementia. All of the 12 NPI domains were rated for symptom frequency from 1 (occasionally) to 4 (very frequently), symptom severity from 1 (mild) to 3 (severe), and caregiver burden from 0 (none) to 5 (extremely) [25].

### 2.4. Assessment of Disease Severity and Cognitive Function

The global severity of dementia was assessed according to the Clinical Dementia Rating (CDR) scale and sum of boxes of the CDR (CDR-SB) [28]. Cognitive functions were assessed with the CASI and the Mini-Mental State Examination (MMSE) modified from the CASI [24]. Motor functions were assessed with motor score of the Unified Parkinson's Disease Rating Scale (UPDRS-m) [29], and all patients were rated under medication. Cognitive tests of all patients were performed by a trained neuropsychologist. Dementia and subtypes of dementia were diagnosed by a consensus meeting composed of three neurologists, one geriatric psychiatrist, and one neuropsychologist. All patients received at least cerebral computed tomography or cerebral magnetic resonance imaging and also a set of blood screening tests for dementia.

### 2.5. Data Analysis

The Chinese version of SPSS 19.0 for Windows (IBM, SPSS Inc., Chicago) was used for statistical analyses. Comparisons between delusional and nondelusional DLB groups in demographic data, CASI, MMSE, motor score of the UPDRS, and composite scores (frequency × severity) of the NPI were analyzed using the independent *t*-test. Gender, CDR, clinical features, clinical history of VRFs, current use of antipsychotics, and current use of antiparkinsonian drugs were analyzed using the chi-square test. To compare the associations of clinical features, cognitive performance, neuropsychiatric symptoms, and VRFs between the delusional and nondelusional groups, we used both model 1 analysis (odds ratios (OR) adjusted for age and gender) and model 2 analysis (OR adjusted for age, gender, disease severity according to the CDR, antipsychotics, and antiparkinsonian drugs).

### 2.6. Ethical Considerations

The Committee for Medical Research Ethics of Show Chwan Memorial Hospital reviewed the project, and the Data Inspectorate approved it.

## 3. Results

**Figure 1:**
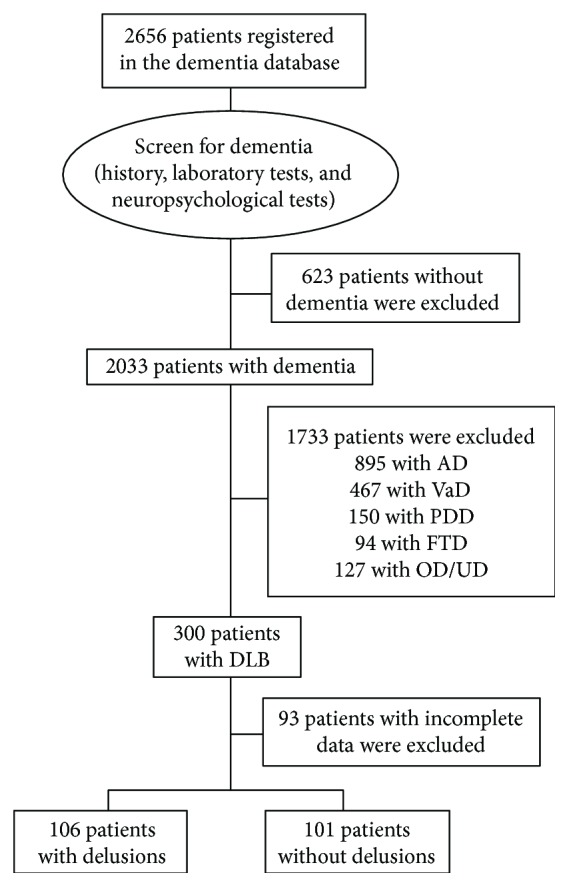


From October 1, 2015, to June 21, 2017, a total of 207 patients who fulfilled the criteria for DLB and had complete data were analyzed. Among them, 106 (51.2%) were delusional and 101 (48.8%) were nondelusional (Figure 1). The delusion of other persons are stealing was the most common (35.3%), followed by delusions of self is in danger (21.3%), house is not his/her home (10.8%), spouse is having an affair (7.2%), family plans to abandon him/her (4.8%), an unwelcome guest is living in the house (2.9%), media persons are in the house (2.9%), and others are not who they claim (1.0%). The frequency of delusions increased as disease severity increased (28.6% in CDR 0.5, 47.5% in CDR 1, and 63.0% in CDR 2–3; *χ*^2^ = 12.776, *p* = 0.002). The severity of delusions among the delusional patients according to the composite score of delusion in the NPI was not different among the CDR groups (4.8 ± 3.7 in CDR 0.5, 4.6 ± 2.1 in CDR 1, and 4.9 ± 2.9 in CDR 2–3; *f* = 0.481, *p* = 0.620).

Comparisons of the demographic data are summarized in Table 1. The delusional group had a significantly higher diagnostic rate of probable DLB (74.5% in the delusional groups versus 60.4% in the nondelusional group, *p* = 0.030), higher disease severity according to CDR stage (*χ*^2^ = 12.776, *p* = 0.002) and CDR-SB (*t* = 3.779, *p* = 0.002), poorer cognitive performance according to the MMSE (*t* = −2.623, *p* = 0.009) and CASI (*t* = −2.629, *p* = 0.009), worse neuropsychiatric symptoms according to the NPI composite score (*t* = 7.144, *p* < 0.001), and higher caregiver burden scale in the NPI (*t* = 10.113, *p* < 0.001).

Comparisons of cognitive performance of each domain in the CASI are summarized in Table 2. The delusional group had poorer performance in the domains of mental manipulation (OR = 0.86, *p* = 0.002) and orientation (OR = 0.93, *p* = 0.008) in model 1 analysis, whereas no cognitive domain was associated with the delusional group in model 2 analysis.

Comparisons of core and suggestive features are summarized in Table 3. The delusional group had a higher frequency of fluctuations (OR = 1.83, *p* = 0.032) and VH (OR = 2.99, *p* < 0.001) in model 1 analysis; however, only VH (OR = 2.31, *p* = 0.019) was significantly higher in the delusional group in model 2 analysis.

A comparison of a history of vascular risk factors demonstrated that the delusional group were significantly less associated with diabetes in both model 1 (OR = 0.36, *p* = 0.002) and model 2 (OR = 0.28, *p* < 0.001) analyses (Table 3). We further analyzed the delusional symptoms that were associated with diabetes after adjusting for age, gender, and disease severity according to the CDR and found that delusions of other persons are stealing (OR = 0.32, *p* = 0.018) and self is in danger (OR = 0.42, *p* = 0.021) were significantly lower in the patients with diabetes.

Comparisons of neuropsychiatric symptoms of each domain in the NPI are summarized in Table 4. The delusional group had higher frequencies of hallucinations (OR = 3.27, *p* < 0.001), agitation (OR = 2.40, *p* = 0.003), anxiety (OR = 2.49, *p* = 0.002), disinhibition (OR = 5.28, *p* < 0.001), irritation (OR = 4.85, *p* < 0.001), and aberrant motor behavior (OR = 2.93, *p* = 0.001) in model 1 analysis. In model 2 analysis, the delusional group had higher frequencies of hallucinations (OR = 2.59, *p* = 0.003), agitation (OR = 1.87, *p* = 0.048), anxiety (OR = 2.64, *p* = 0.002), disinhibition (OR = 4.81, *p* = 0.003), irritation (OR = 4.50, *p* < 0.001), and aberrant motor behavior (OR = 2.36, *p* = 0.010).

## 4. Discussion

In this study, about half (51.2%) of all patients had a delusion, and the delusion of other persons are stealing (35.3%) was the most common, followed by delusion of self is in danger (21.3%). These findings are consistent with the results from most of the previous studies on DLB [10–13] and also clinical study on AD [8]. The finding of higher frequency of delusions in more severe dementia is probably because delusions are highly associated with the ability of source memory monitoring, and this ability is gradually deteriorated as disease progresses [30]. In this study, although only a cognitive screening tool was used to study the association of cognitive functions with delusions [24], our patients with DLB and delusions had poorer cognitive function, especially in the domain of mental manipulation, which is also regarded to be an executive function [31]. Studies on the mechanism and interaction of delusions with cognition had demonstrated that delusions are highly associated with cognitive impairment and especially with impairments in source monitoring [30] and are regarded to involve source memory and executive functions [30, 32]. A recent study on the interaction of cognitive functions and delusions in patients with AD also reported that psychosis is influenced by executive function [33]. In addition, relationships between behavioral syndromes and cognitive domains in patients with AD showed that psychosis was significantly associated with impaired working memory [33]. In previous studies of DLB and according to the consensus criteria, cognitive impairments in the domains of executive function, visuospatial function, and attention in patients with DLB have been noted in the early stage of disease [1, 3]. Therefore, it is reasonable to find a high frequency of delusions in patients with DLB.

Previous studies have shown that neuropsychiatric symptoms in patients with DLB are more severe and more frequent than in other types of dementia [9–14]. These symptoms are salient in the early stage of DLB, and they are manifested as delusions, visual hallucinations, REM sleep behavior disorder, and depression [1, 3]. Our patients with mild DLB had a high frequency of delusions (22.7% with CDR 0.5 and 39.5% with CDR 1), which is consistent with our previous study on delusions in different stages of AD [8]. The current study also demonstrated that the delusional patients generally had more severe neuropsychiatric symptoms and were associated with a higher frequency of hallucinations, agitation/aggression, anxiety, irritation, and aberrant motor behavior.

Previous studies on the association between vascular risk factors and degenerative and/or vascular disorders have focused on controlling risk factors in midlife to prevent morbidity and mortality in late life. In general, these factors are regarded to be important risk factors for both small and large vessel diseases, and most of the vascular risk factors, including diabetes, in midlife have been associated with increased neurodegenerative dementia and vascular dementia in late life [21, 23]. However, the association between the incidence of dementia or cognitive decline and diabetes in late life is still controversial. The contribution of vascular risk factors has seldom been studied in patients with DLB. A previous study on the risk factors for DLB compared to the risk factors for AD showed no differences in stroke or diabetes between the two groups [22]. A novel finding of the current study is that the patients with DLB comorbid with diabetes had a lower frequency of delusions. A possible explanation for this finding is that, similar to findings from a study of animals with diabetes, levels of muscarinic acetylcholine receptors (mAChRs) subtype M1 are decreased in the cerebral cortex of patients with diabetes [34]. The M1 subtype of mAChRs is the most abundant type in the human cerebral cortex and hippocampus [35]. In general, M1 immunoreactivity is markedly reduced in the brains of patients with AD and DLB [34]. However, in a study of autopsy cases, Ballard et al. reported that delusions in patients with DLB are associated with elevated M1 binding in Brodmann area 36 [12]. Another study found decreased levels of total muscarinic and muscarinic M1 receptors in animals with diabetes [34]. Based on these findings, we proposed that patients with DLB comorbid with diabetes may have decreased levels of M1 receptors in the brain, which may lead to a lower frequency of delusions. Further studies are warranted to clarify the pathophysiology and causal relationship among diabetes, antidiabetes drugs, and delusions in patients with DLB.

In conclusion, delusions and other neuropsychiatric symptoms were evaluated in a relatively large sample of patients with DLB in this study. We found that the frequency of delusions increased as the severity of dementia increased in patients with DLB. We use multidimensional analysis of the associated factors of delusions and found that the patients with DLB and delusions had a poorer cognitive function and more severe neuropsychiatric symptoms. The novel finding of this study is that the patients with DLB comorbid with diabetes had a lower frequency of delusions.

## Figures and Tables

**Table 1:** Demographic and background characteristics between the delusional and nondelusional patients with DLB.

	Delusional	Nondelusional	*t*/*χ*^2^	*p*
*N*	106	101		
Gender, male/female	53/53	62/39	2.716	NS
Age, years (SD, range)	78.9 (6.8, 62–91)	77.6 (6.8, 51–90)	−1.301	NS
Education, years (SD, range)	5.8 (5.2, 0–18)	6.6 (5.0, 0–19)	−1.047	NS
Duration, years (SD, range)				
Dementia	3.0 (3.0, 0–20)	2.8 (2.8, 0–15)	0.358	NS
Parkinsonism	3.0 (3.0, 0–20)	2.6 (2.3, 0–10)	1.019	NS
Psychiatric disorder	4.0 (5.1, 0–40)	3.2 (3.6, 0–25)	1.251	NS
RBD	5.0 (9.5, 0–50)	5.5 (11.0, 0–50)	−0.225	NS
DLB probable/possible	79/27	61/40	4.719	0.030
CDR 0.5/1/2–3	10/38/58	25/42/32	12.776	0.002
CDR-SB (SD, range)	9.3 (3.3, 1.5–17.0)	7.5 (3.8, 1.5–16.0)	3.779	<0.001
MMSE (SD, range)	14.0 (7.0, 0–29)	16.9 (8.0, 0–29)	−2.623	0.009
CASI (SD, range)	44.7 (23.1, 0–95)	53.7 (25.9, 0–94)	−2.629	0.009
NPI (SD, range)	34.3 (14.6, 3–77)	19.4 (15.4, 0–79)	7.144	<0.001
NPI burden (SD, range)	17.9 (6.7, 0–32)	9.1 (5.8, 0–25)	10.113	<0.001
UPDRS-m (SD, range)	17.7 (11.3, 0–63)	18.1 (11.4, 0–48)	−0.279	NS
Antipsychotics, *n* (%)	22 (20.8%)	12 (11.9%)	2.967	NS
Antiparkinsonian, *n* (%)	41 (38.7%)	42 (41.6%)	0.182	NS
LED (SD, range)	146 (237, 0–1050)	181 (276, 0–1158)	0.977	NS

NS: not significant; DLB: dementia with Lewy bodies; RBD: REM sleep behavior disorder; psychiatric disorder: psychosis or mood disorders; DLB probable/possible: diagnosis of probable DLB/possible DLB; CDR: Clinical Dementia Rating scale; CDR-SB: sum of boxes of CDR; MMSE: Mini-Mental State Examination; CASI: Cognitive Abilities Screening Instrument; NPI: total score of the 12-domain Neuropsychiatric Inventory; NPI burden: total caregiver burden scale in the NPI; UPDRS-m: motor score of the Unified Parkinson's Disease Rating Scale; antipsychotics: current using antipsychotics; antiparkinsonian: current use of antiparkinsonian agents; LED: levodopa equivalent dose, mg/day.

**Table 2:** Two models of risk estimates (odds ratios) for cognitive domains in CASI between the delusional and nondelusional patients with DLB.

Features	Mean (SD, range)		Model 1		Model 2	
	Delusional	Nondelusional	OR (95% CI)	*p*	OR (95% CI)	*p*
*N*	106	101				
Remote memory	6.6 (3.3, 0–10)	7.3 (3.2, 0–10)	0.95 (0.87–1.04)	NS	1.05 (0.94–1.17)	NS
Recent memory	4.2 (3.4, 0–12)	5.4 (3.7, 0–12)	0.92 (0.85–1.00)	NS	1.00 (0.90–1.10)	NS
Attention	5.2 (2.2, 0–8)	5.4 (2.2, 0–8)	0.98 (0.86–1.12)	NS	1.09 (0.94–1.27)	NS
Mental manipulation	2.5 (2.9, 0–10)	4.0 (3.5, 0–10)	**0.86 (0.77–0.95)**	**0.002**	0.91 (0.81–1.03)	NS
Orientation	6.7 (4.8, 0–18)	9.0 (6.0, 0–18)	**0.93 (0.88–0.98)**	**0.008**	0.99 (0.92–1.06)	NS
Abstract thinking	4.1 (2.8, 0–11)	4.8 (3.0, 0–12)	0.95 (0.85–1.05)	NS	1.04 (0.91–1.08)	NS
Language	6.8 (3.1, 0–10)	7.1 (3.0, 0–10)	0.98 (0.89–1.07)	NS	1.08 (0.96–1.20)	NS
Draw	4.8 (3.8, 0–10)	5.9 (3.9, 0–10)	0.94 (0.87–1.01)	NS	1.01 (0.92–1.10)	NS
Animal naming (verbal fluency)	4.0 (3.0, 0–10)	4.8 (3.3, 0–10)	0.94 (0.86–1.03)	NS	1.02 (0.92–1.14)	NS

DLB: dementia with Lewy bodies; CASI: Cognitive Abilities Screening Instrument; NS: not significant. The odds ratio (OR) and 95% confidence interval (CI) were calculated with the nondelusional group as reference. Model 1 ORs were adjusted for age, gender, and education; model 2 ORs were adjusted for age, gender, education, disease severity, antipsychotics, and antiparkinsonian agents.

**Table 3:** Two models of risk estimates (odds ratios) for core and suggestive features between the delusional and nondelusional patients with DLB.

Features	*N* (%)		Model 1		Model 2	
	Delusional	Nondelusional	OR (95% CI)	*p*	OR (95% CI)	*p*
*N*	106	101				
Clinical features						
Fluctuation	62 (58.5%)	44 (43.6%)	**1.83 (1.05–3.17)**	**0.032**	1.52 (0.84–2.73)	NS
Visual hallucinations	65 (61.3%)	35 (34.7%)	**2.99 (1.70–5.27)**	**<0.001**	**2.26 (1.21–4.21)**	**0.010**
Parkinsonism	96 (90.6%)	87 (86.1%)	1.54 (0.65–3.66)	NS	1.26 (0.49–3.25)	NS
RBD	44 (41.5%)	45 (44.6%)	0.88 (0.51–1.53)	NS	1.18 (0.66–2.12)	NS
Neuroleptic sensitivity^∗^	12 (11.3%; 54.5%)	7 (6.9%; 58.3%)	1.71 (0.65–4.55)	NS	1.72 (0.61–4.83)	NS
Vascular risk factors						
Hypertension	52 (49.1%)	45 (44.6%)	1.10 (0.63–1.92)	NS	1.17 (0.65–2.09)	NS
Diabetes	21 (19.8%)	40 (39.6%)	**0.36 (0.19–0.68)**	**0.002**	**0.28 (0.14–0.56)**	**<0.001**
Coronary artery disease	7 (6.6%)	7 (6.9%)	0.93 (0.31–2.81)	NS	0.82 (0.26–2.60)	NS
Hyperlipidemia	4 (3.8%)	5 (5.0%)	0.84 (0.22–3.26)	NS	0.80 (0.20–3.21)	NS
Arrhythmia	9 (8.5%)	13 (12.9%)	0.67 (0.27–1.67)	NS	0.55 (0.21–1.46)	NS
Cerebrovascular disease	16 (15.1%)	19 (18.8%)	0.58 (0.33–1.02)	NS	0.63 (0.29–1.38)	NS

DLB: dementia with Lewy bodies; RBD: REM sleep behavior disorder; NS: not significant. The odds ratio (OR) and 95% confidence interval (CI) were calculated with the nondelusional group as reference. ^∗^Severe neuroleptic sensitivity (among all patients; among those who had ever used antipsychotics). Model 1 ORs were adjusted for age and gender. Model 2 ORs were adjusted for age, gender, disease severity, antipsychotics, and antiparkinsonian agents.

**Table 4:** Two models of risk estimates (odds ratios) for neuropsychiatric symptoms in the NPI between the delusional and nondelusional DLB groups.

Neuropsychiatric symptoms	Mean (SD, range)		Model 1		Model 2	
	Delusional	Nondelusional	OR (95% CI)	*p*	OR (95% CI)	*p*
*N*	106	101				
Hallucinations	71 (67.0%)	37 (36.6%)	**3.27 (1.83–5.85)**	**<0.001**	2**.59 (1.39–4.80)**	**0.003**
Agitation	52 (49.1%)	30 (29.7%)	**2.40 (1.34–4.30)**	**0.003**	**1.87 (1.01–3.48)**	**0.048**
Depression	76 (71.7%)	65 (64.4%)	1.31 (0.72–2.38)	NS	1.19 (0.64–2.22)	NS
Anxiety	72 (67.9%)	45 (44.6%)	**2.49 (1.40–4.43)**	**0.002**	**2.64 (1.45–4.81)**	**0.002**
Euphoria	2 (1.9%)	2 (2.0%)	1.22 (0.14–9.02)	NS	0.62 (0.77–4.96)	NS
Apathy	72 (67.9%)	59 (58.4%)	1.58 (0.88–2.82)	NS	1.26 (0.68–2.32)	NS
Disinhibition	24 (22.6%)	5 (5.0%)	**5.28 (1.91–14.44)**	**<0.001**	**4.81 (1.71–13.53)**	**0.003**
Irritation	62 (58.5%)	24 (23.8%)	**4.85 (2.62–8.99)**	**<0.001**	**4.50 (2.39–8.46)**	**<0.001**
Aberrant motor behavior	50 (47.2%)	23 (22.8%)	**2.93 (1.59–5.38)**	**0.001**	**2.36 (1.23–4.51)**	**0.01**
Sleep	91 (85.8%)	78 (77.2%)	1.72 (0.83–3.55)	NS	1.56 (0.74–3.30)	NS
Eat/appetite	47 (44.3%)	30 (29.7%)	1.54 (0.87–2.74)	NS	1.36 (0.75–2.46)	NS

DLB: dementia with Lewy bodies; NPI: Neuropsychiatric Inventory; NS: not significant. The odds ratio (OR) and 95% confidence interval (CI) were calculated with the nondelusional group as reference. Model 1 ORs were adjusted for age and gender; model 2 ORs were adjusted for age, gender, disease severity, antipsychotics, and antiparkinsonian agents.

## Data Availability

The data used to support the findings of this study are available from the corresponding author upon request.

## Additional Points

*Significant Outcomes*. (1) Neuropsychiatric symptoms were evaluated in a relatively large sample of patients with dementia with Lewy bodies (DLB). (2) We use multidimensional analysis of the associated factors of delusions in patients with DLB. (3) A novel finding is that patients with DLB comorbid with diabetes had fewer delusions. *Limitations*. (1) This study was conducted in 3 hospitals in Taiwan. Therefore, the findings may not be generalizable to all patients with DLB. (2) The comparison of associated factors between the delusional and nondelusional patients with DLB in this study was cross-sectional. Therefore, causal relationships of the factors and dementia could not be ascertained. (3) Because of a lack of measurable data including the glucose level, glycated hemoglobin level, blood pressures, and medications on vascular risk factors and the associated medications of the patients, further studies are needed to evaluate the contribution of these factors on the presentation of delusions in patients with DLB. (4) Because less than 20% of our participants had received dopamine transporter uptake imaging, this may have resulted in a lower diagnostic rate for probable DLB in this study.
